# *Euglena gracilis*-derived β-1,3-glucan as a growth substrate for *Lactococcus lactis—*a snapshot of accompanying changes in transcriptomic and metabolomic profiles

**DOI:** 10.3389/fmicb.2026.1894637

**Published:** 2026-09-25

**Authors:** Hidehiko Kato, Jens Mortansson Jelstrup Nolsøe, Hetron Mweemba Munang'Andu, Viswanath Kiron

**Affiliations:** Faculty of Biosciences and Aquaculture, Nord University, Bodø, Norway

**Keywords:** algal beta glucans, bacterial metabolites, fermentation, lactic acid bacteria, metabolic reprogramming, paramylon

## Abstract

β-1,3-glucan from *Euglena gracilis* is an emerging microalgal prebiotic. However, its potential to reprogram the metabolic profile of lactic acid bacteria has not been reported previously. In the present study, the effects of insoluble β-1,3-glucan on growth dynamics, gene expression, and the extracellular metabolite profile of *Lactococcus lactis* DSM20175 were investigated using transcriptomic and untargeted metabolomic approaches. Real-time growth monitoring combined with flow cytometric cell enumeration showed that β-1,3-glucan supplementation significantly increased both growth rate and final cell density compared with a control group that did not receive any supplementation. Transcriptomic analysis showed a broad downregulation of genes associated with carbon catabolite repression, Rgg/GadR/MutR-family regulators, surface proteins, and membrane transporters, likely indicating an earlier transition from exponential to stationary phase physiology. Untargeted metabolomics revealed extensive differential extracellular metabolite secretion, including shared increases in signature metabolites of fermentation, as well as β-glucan-specific elevation of aromatic and tryptophan-derived metabolites. These multi-omics data revealed that the β-1,3-glucan from *E. gracilis* functions as a growth substrate, accelerating the growth-phase transition and reshaping metabolic pathways of *L. lactis* DSM20175. This baseline information has potential implications for synbiotic formulation and industrial fermentation processes.

## 1 Introduction

Lactic acid bacteria (LAB) are naturally present in diverse terrestrial and aquatic environments where they perform a variety of functions (Lugli et al., 2025; Mariom et al., 2025; Wang et al., 2021). Under suitable conditions, they adopt flexible fermentative pathways to generate a range of antimicrobial and bioactive metabolites, including lactic acid, acetate, polyamines, and various phenolic and aromatic compounds, which contribute to microbial stability and the maintenance of ecosystem balance (Abouelela and Helmy, 2024; Alves et al., 2023).

*Lactococcus lactis*, a lactic acid-producing bacterium, is considered a promising candidate for therapeutic applications due to its ability to synthesize several specific bacteriocins (Holo et al., 1991; Iwatani et al., 2007; Zendo et al., 2006) and host-relevant metabolites upon utilizing available substrates, particularly fermentable carbohydrates (Buksa et al., 2025; Navale et al., 2023). Given its fermentative abilities, its association with the host environment, the genetic basis underlying its metabolic functions, and the established role of non-digestible carbohydrates in host health, it is imperative to advance our understanding of how carbohydrates influence the metabolic outputs and functional potential of *L. lactis*. Such knowledge may enable the optimized use of both prebiotics and probiotics to improve gastrointestinal functions in humans.

Among complex dietary carbohydrates, β-glucans have attracted substantial attention as functional polysaccharides. Linear forms are derived from diverse sources, including fungi, bacteria, microalgae, and seaweed. In the microalga *Euglena gracilis*, they occur as the storage polysaccharide paramylon (β-1,3-glucan), which has been promoted as a health food resource (Dai et al., 2022), possibly due to its ability to act as a ligand for the β-glucan receptor Dectin-1, expressed on intestinal epithelial and immune cells. Co-administration of this form of β-1,3-glucan with LAB may confer health benefits in humans, even though most characterized LAB lack genes encoding exo-/endo-β-1,3-glucanases and are unable to depolymerize the polysaccharides directly. This effect may be mediated indirectly through cross-feeding interactions with other microorganisms capable of degrading complex polysaccharides. Interestingly, *E. gracilis* biomass containing β-1,3-glucan has been reported to stimulate the growth of commensal *Faecalibacterium*, thereby modulating human colonic microbiota (Nakashima et al., 2021). Additionally, these β-glucans have been shown to promote the growth of *Lactobacillus, in vitro* (Bockwoldt et al., 2022; Buksa et al., 2025).

Understanding such complex prebiotic-probiotic interactions requires a multi-omics approach, which can delineate associated metabolic pathways and information regarding the production of functional metabolites arising from synergistic or complementary microbial interactions (Cui et al., 2024; Wang et al., 2021). Characterizing *L. lactis* responses to β-1,3-glucans through transcriptomic and metabolomic profiling enables evidence-based modeling of substrate-induced changes in probiotic phenotypes and mechanisms. The present *in vitro* study investigated changes in the transcriptome and metabolome of *L. lactis* DSM20175 following exposure to *E. gracilis*-derived β-1,3-glucan. This work provides a broader understanding of how β-1,3-glucan exposure modulates the physiology of the studied LAB, including shifts in gene expression and metabolite production relevant to growth and metabolism.

## 2 Materials and methods

### 2.1 Bacterial strains and culture conditions

*Lactococcus lactis* DSM20175 was obtained from the German culture collection (DSMZ) in lyophilized form and revitalized upon arrival. Throughout the experiment, *L. lactis* was cultured in Brain Heart Infusion medium (BHI; Merck KGaA, Darmstadt, Germany) supplemented with 0.5 g/L cysteine-HCl and 0.1 % porcine gastric mucin (Sigma-Aldrich, Massachusetts, USA). Cysteine-HCl was added as a reducing agent to create an oxygen-limited environment, and porcine gastric mucin was required to mimic mucin-rich gut environment. A 100 mL flask containing the culture medium was inoculated and sealed with a Versilic^®^ silicone cap (Thermo Fisher Scientific, Loughborough, UK) inside an anaerobic workstation (Don Whitley Scientific, Bingley, UK) supplied with a gas mixture (5 % H_2_, 5 % CO_2_, 90 % N_2_). The culture flasks were incubated anaerobically in the dark at 37 °C on an orbital shaker (VWR, Pennsylvania, USA) at 110 rpm. Culture flasks containing only BHI medium were defined as the negative control, while flasks with 1 mg/mL of insoluble β-1,3-glucan derived from *Euglena gracilis* (Sigma-Aldrich, Massachusetts, USA; referred to as BG) and the probiotic (approximately 1.0 × 10^5^ of *L. lactis* cells per 100 μL) served as the treatment group. Flasks containing the same number of *L. lactis* cells in the BHI medium were included as the probiotic control, whereas flasks containing the BG and BHI medium were identified as the BG control group.

### 2.2 Real-time bacterial growth monitoring

Real-time growth rate was measured and recorded every 60 sec using Cell Growth Quantifier (CGQ)^®^ sensors (Scientific Bioprocessing, Baesweiler, Germany), which emit a combination of wavelengths at 521 nm and 940 nm, subsequently capturing backscattered photons using a photodiode, and convert the light intensity into a weak electric current for calculation of the cell density. At the late exponential phase, after 5.5 h of incubation, samples were collected for cell enumeration, transcriptomics, and untargeted metabolomic analysis.

### 2.3 Counting bacterial cells with flow cytometry

For bacterial cell enumeration, 100 μL of *L. lactis* culture was collected from each sample and diluted to a final volume of 1 mL with deionized water. Cell counts (cells/mL) were determined using a SH800S cell sorter (Sony Europe, Weybridge, UK) equipped with a 100 μm microfluidic sorting chip. A high affinity nucleic acid stain, SYTO™ BC dye from the Bacteria Counting Kit (Thermo Fisher Scientific, Loughborough, UK), was added to each sample to enumerate bacterial counts accurately by flow cytometry. The gating strategy used to identify the stained cells is presented in Figure 1. The formula for bacterial enumeration, provided in the instructions accompanying the kit, is presented below.


$$\begin{array}{l} Number\;of\;bacteria\left(\frac{cells}{mL}\right)\\ =\frac{stained\;cell\;\left(gate\;C\right)}{microsphere\;beads\;\left(gate\;B\right)\times 10^{-6}}\times dilution\;factor\left(10^{2}\right). \end{array}$$

**Figure 1:**
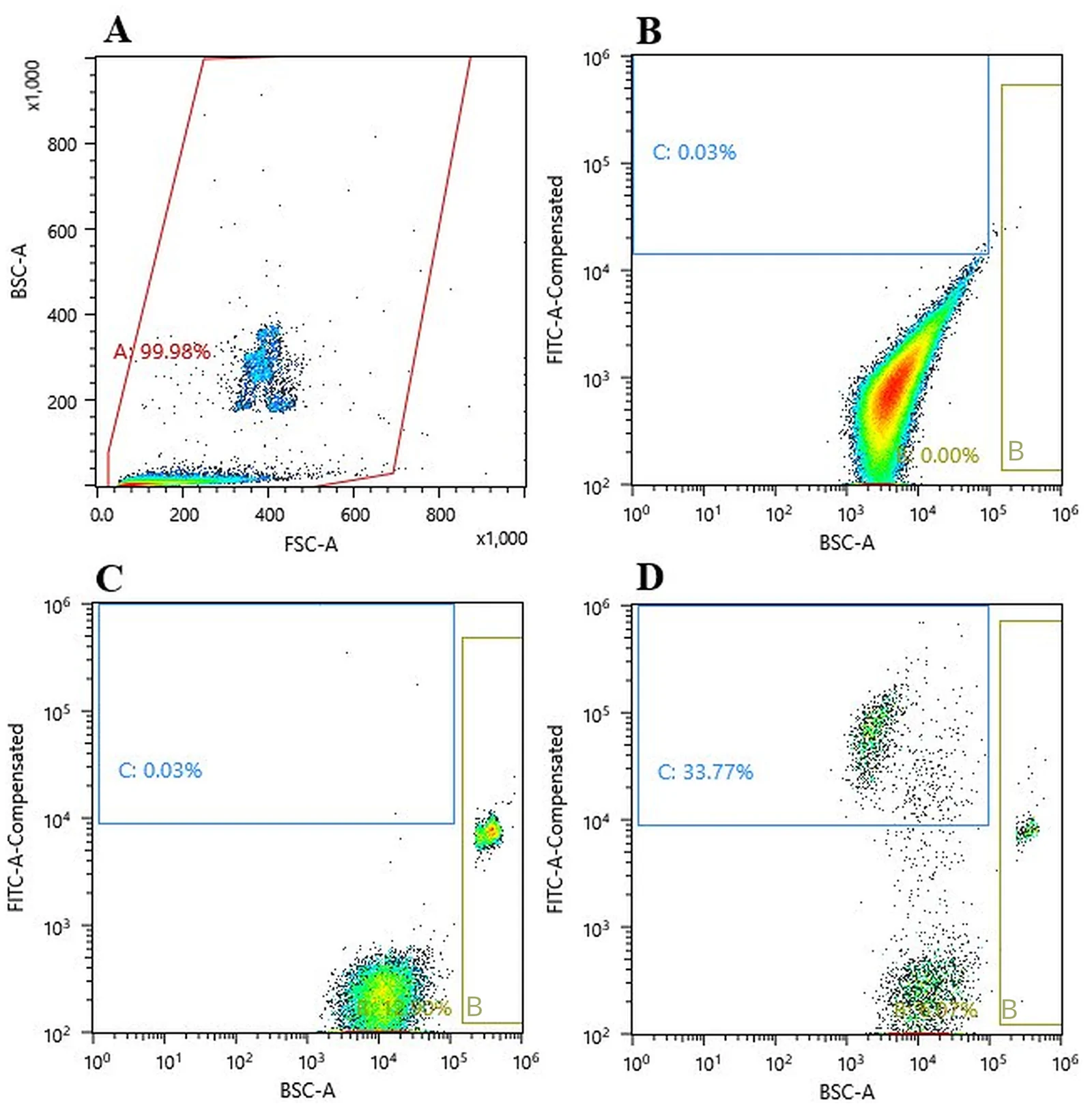
Visualization of the gating strategy to determine the counts of *Lactococcus lactis*. **(A)** Gating of all the captured events (red gate A indicates all the cells, microsphere beads, and β-1,3-glucan population); **(B)** Gating to identify the unstained cells (yellow gate B indicates population of microsphere beads, and blue gate C indicates population of stained cells); **(C)** Gating to separate β-1,3-glucan and microsphere beads without stained cells; **(D)** Gating to identify stained cells (blue gate C), β-1,3-glucan, and microsphere beads (yellow gate B).

The cell counts of four biological replicates from each group were statistically compared by the Mann–Whitney *U* test using R (version 4.5.2).

### 2.4 Transcriptomics and metabolomics

Based on the growth data, the subsequent transcriptomic and metabolic studies were performed only on samples from the Probiotic control (BHI + *L. lactis*) and the treatment group (BHI + *L. lactis* + BG).

#### 2.4.1 Transcriptomics data generation and analysis

Cultured *L. lactis* was collected from each flask (4 flasks/biological replicates for both the probiotic control and the treatment group) into a sterile 1.5 mL Eppendorf tube. The samples were centrifuged at 13,000 × *g* for 1 min at 4 °C. Subsequently, to avoid cross-contamination, 1 mL of supernatant was transferred to a new 1.5 mL Eppendorf tube, flash-frozen in liquid nitrogen, and stored at −80 °C for metabolomics analysis (section 2.4.2). The remaining supernatant was carefully removed, and the resulting pellet (bacterial biomass) was immediately frozen in liquid nitrogen. Thereafter, 100 μL of phenol-chloroform-isoamyl alcohol (25:24:1, v/v/v) was added to the frozen sample. The samples were homogenized with 0.1 mm glass beads using a Precellys evolution homogenizer (Bertin Technologies, Montigny-le-Bretonneux, France) at 5,000 rpm for two 30-sec cycles, with 30-sec intervals between cycles. The homogenized samples were centrifuged at 15,000 × *g* for 1 min at 4 °C, and then the instructions for the QIAGEN AllPrep DNA/RNA Kit (QIAGEN, Venlo, The Netherlands) were followed for RNA isolation. Purified RNA was eluted in RNase-free water, and its quality was assessed using NanoDrop One^®^ (Thermo Fisher Scientific, MI, USA) and an Agilent 2200 TapeStation system with RNA ScreenTape (Agilent Technologies, Waldbronn, Germany) to confirm that the RNA integrity number (RIN) was >6.0.

Prokaryotic RNA library preparation and sequencing were performed by NovoGene Corporation (UK) on an Illumina high-throughput sequencing platform (NovaSeq X) using a 150 bp paired-end sequencing strategy. Adapter sequences were removed from raw reads, and reads containing more than 10 % ambiguous bases (N) or more than 50 % low-quality bases (Q score ≤ 5) were filtered. The reference genome sequence for *L. lactis* (GCF_003176835.1_ASM317683v1_genomic.fna) and its corresponding gene annotation file (GCF_003176835.1_ASM317683v1_genomic.gff) were obtained from NCBI. Ribosomal RNA sequences were removed using BBDuk (Bushnell, 2025). RNA-Seq reads were aligned to the reference genome using Bowtie2 with the paired-end option (Langmead and Salzberg, 2012). Aligned reads were assigned to genomic coding sequences (CDSs) and quantified using the “featureCounts” function in the Subread toolkit (Liao et al., 2019). Read-count data from all samples were analyzed in R (version 4.5.2). Differentially expressed genes were determined using DESeq2 (Love et al., 2014) by considering the log_2_ fold change (Log_2_FC) and the *p*-values. Benjamini-Hochberg correction was applied to calculate the adjusted *p*-values for each gene. Gene ontology (GO) and kyoto encyclopedia of genes and genomes (KEGG) orthology (KO) enrichment analyses were performed separately for up- and downregulated genes using Fisher's exact test, implemented in topGO (v2.62.0; Alexa and Rahnenfuhrer, 2025) and clusterProfiler (v4.18.4; Xu et al., 2024), respectively. For exploratory GO/KEGG pathway analysis, a threshold (*p* < 0.1) was used, while retaining the full set of annotated genes of *L. lactis* as the background. Gene set enrichment analysis (GSEA) was also performed with all the expressed genes and their log_2_FC using clusterProfiler (v4.18.4). KO terms for the *L. lactis* were annotated using protein sequences (GCF_003176835.1_ASM317683v1_protein.faa) with the BlastKOALA tool, and the KEGG pathways specific to *L. lactis* were retrieved from the KEGG database using KEGGREST (v1.52.2; Tenenbaum, 2026). Raw reads were deposited in the NCBI Sequence Read Archive (SRA) under the accession number PRJNA1418359.

#### 2.4.2 Untargeted metabolomics analysis

The supernatants collected as described in section 2.4.1 were used for the untargeted metabolomics analysis (4 biological replicates for the probiotic control and treatment groups). Sample preparation and bacterial metabolite profiling were performed by Cmbio Corporation (Copenhagen, Denmark). The semi-polar metabolite profiling was performed using a UHPLC system (Vanquish, Thermo Fisher Scientific, UK) coupled with a high-resolution quadrupole-orbitrap mass spectrometer (Orbitrap Exploris 240 MS, Thermo Fisher Scientific, UK). Each compound was assigned to one of three annotation-confidence levels. Compounds identified with the highest confidence were classified as annotation level 1, based on accurate mass, MS/MS spectra, and known retention time. Annotation level 2 was further divided into two sublevels: level 2a, based on accurate mass and known retention time, and level 2b, based on accurate mass and MS/MS spectra measured with high resolution Orbitrap ESI-MS and matched against mzCloud (Thermo Fisher Scientific), MassBank of North America (UC Davis), and the European MassBank (Helmholtz Center for Environmental Research Leipzig). Annotation level 3 was assigned based on library searches using accurate mass and elemental composition only. For the univariate analysis, raw data were normalized using probabilistic quotient normalization (PQN), a robust technique for addressing dilution effects and concentration variability, thereby reducing variation and facilitating the interpretation of metabolomics data (Dieterle et al., 2006). Following PQN, Box-Cox power transformation was applied using the R package caret (v7.0) to improve the data normality. After confirming normality and variance homoscedasticity using the Shapiro–Wilk test and Levene's test, unpaired *t*-tests were performed, and Benjamini-Hochberg correction was applied to calculate adjusted *p*-values for each compound. These statistical tests were performed in R (version 4.5.2). For the KEGG pathway analysis, MetaboAnalyst 6.0 was used to annotate the metabolites, and the KEGG pathways for the metabolites of *L. lactis* were retrieved using KEGGREST (v1.52.2). The clusterProfiler (v4.18.4) was used to perform both over-representation analysis (ORA) and GSEA for interpreting the metabolite abundance data (Subramanian et al., 2005). A threshold of *p* < 0.1 was used for the analysis. Raw peak area data were deposited in Zenodo under DOI 10.5281/zenodo.21970163.

## 3 Results

### 3.1 Influence of β-1,3-glucans on the growth rate of *L. lactis*

*L. lactis* DSM20175 cultured in BHI medium without β-1,3-glucan supplementation (probiotic control group) exhibited an average backscatter intensity of 242 (arbitrary unit), corresponding to the flow cytometry counts of approximately 8 × 10^7^ cells/mL after the cultivation period (Figure 2). In contrast, supplementation of the BHI medium with β-1,3-glucan (treatment group) resulted in a significantly higher backscatter intensity compared to the control group. The average backscatter intensity for the treatment group reached 440, corresponding to approximately 1.2 × 10^8^ cells/mL at the end of the experimental period. The treatment group showed a significantly increased growth rate and final cell density relative to the probiotic control group (*p* < 0.05) (Supplementary Table 1).

**Figure 2:**
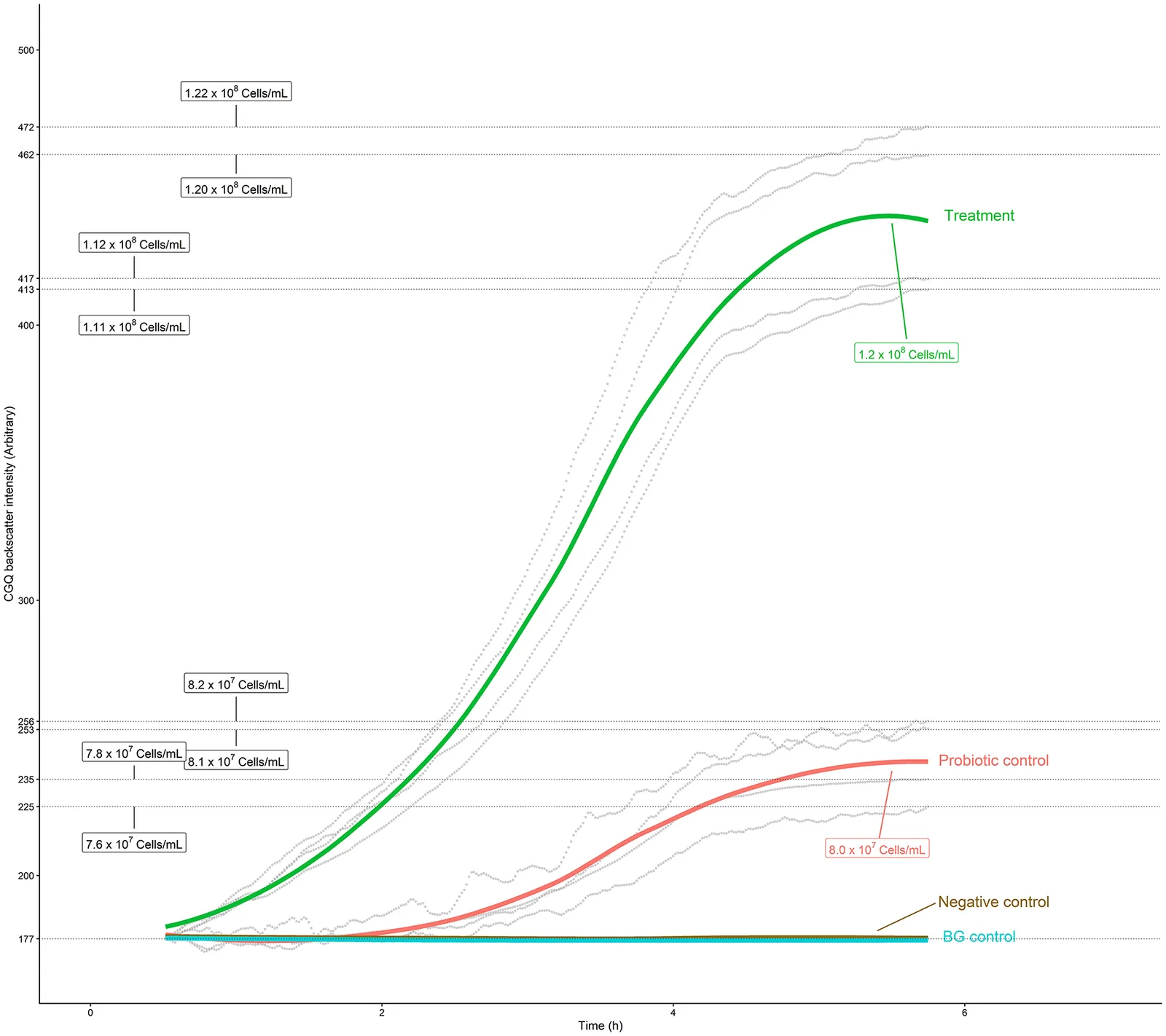
Growth curves showing the average backscatter intensity for the probiotic control and the treatment group. BG control: indicating that there was no increase in the backscatter intensity by BG itself. Negative control: containing only the culture media (BHI). Gray curves represent biological replicates (*n* = 4 flasks per group), while horizontal lines indicate the corresponding cell counts for each sample.

### 3.2 Genes of *L. lactis* that were altered upon β-glucan supplementation

Sequenced reads were obtained through bulk RNA-Seq of cultured *L. lactis*. Four replicate samples from each group [Probiotic control (BHI + *L. lactis*) and the treatment group (BHI + *L. lactis* + BG)] generated between 8,849,276 and 56,813,234 reads per sample. Alignment rates to the *L. lactis* reference genome ranged from 87.0 to 88.7 %, and 86.3 to 88.1 % of the aligned genes were successfully annotated and quantified for the downstream analysis (Supplementary Tables 2 and 3). Comparison between the probiotic control and the treatment groups identified 26 significantly differentially expressed genes (DEGs; *p* < 0.05), of which 23 were downregulated and 3 were upregulated. Using a more relaxed threshold (*p* < 0.1), the number of DEGs increased from 26 to 32 (Figure 3). Significantly upregulated DEGs in the treatment group included the transcription factor *rpoD* (raw *p* = 7 × 10^−4^, adjusted *p* = 0.05), and the DNA replication-associated gene *dnaG* (raw *p* = 0.001, adjusted *p* = 0.07), and genes encoding membrane-associated transporters, including “heavy metal translocating *P*-type ATPase” (raw *p* = 5 × 10^−4^, adjusted *p* = 0.04) and “MFS transporter 7” (raw *p* = 6 × 10^−4^, adjusted *p* = 0.05). Most of the significant DEGs were downregulated, including transcriptional regulators such as *ccpA* (raw *p* = 7 × 10^−5^, adjusted *p* = 8 × 10^−3^), “LysR family transcriptional regulator 2” (raw *p* = 5 × 10^−4^, adjusted *p* = 0.04), and “Rgg/GadR/MutR-family transcriptional regulators five and six” (raw *p* = 7 × 10^−10^, adjusted *p* = 4 × 10^−7^ and raw *p* = 4 × 10^−5^, adjusted *p* = 5 × 10^−3^), as well as multiple membrane-associated transporters, including “amino acid ABC transporter permease 1” (raw *p* = 2 × 10^−11^, adjusted *p* = 3 × 10^−8^), *oppD* (raw *p* = 2 × 10^−5^, adjusted *p* = 3 × 10^−3^), and *lmrP* (raw *p* = 2 × 10^−5^, adjusted *p* = 3 × 10^−3^). Strongly downregulated genes also included those encoding surface proteins such as “WxL domain-containing protein 2” (raw *p* = 1 × 10^−3^, adjusted *p* = 0.08) and “leucine-rich repeat (LRR) domain-containing protein” (raw *p* = 3 × 10^−7^, adjusted *p* = 1 × 10^−5^). In addition to the widespread downregulation, several ribosomal protein genes showed a non-significant tendency toward lower expression, including *rpsN, rpmB, rplC, rplD, rplE, rplJ, rplS, rplM, rplK, rplR, rpsJ, rpmG, rpsB, rpsH, rplB, rplT, rpsD, rplL, and rpsO* (raw *p* = 0.03–0.6, adjusted *p* = 0.4–0.9; Supplementary Table 4). Genes for carbohydrate and metal transport, including “PTS sugar transporter subunit IIA 2” (raw *p* = 0.03, adjusted *p* = 0.4), *feoB* (raw *p* = 6 × 10^−3^, adjusted *p* = 0.2), and “cation diffusion facilitator family transporter 2” (raw *p* = 5 × 10^−3^, adjusted *p* = 0.2), also showed a non-significant tendency toward higher expression (Supplementary Table 5).

**Figure 3:**
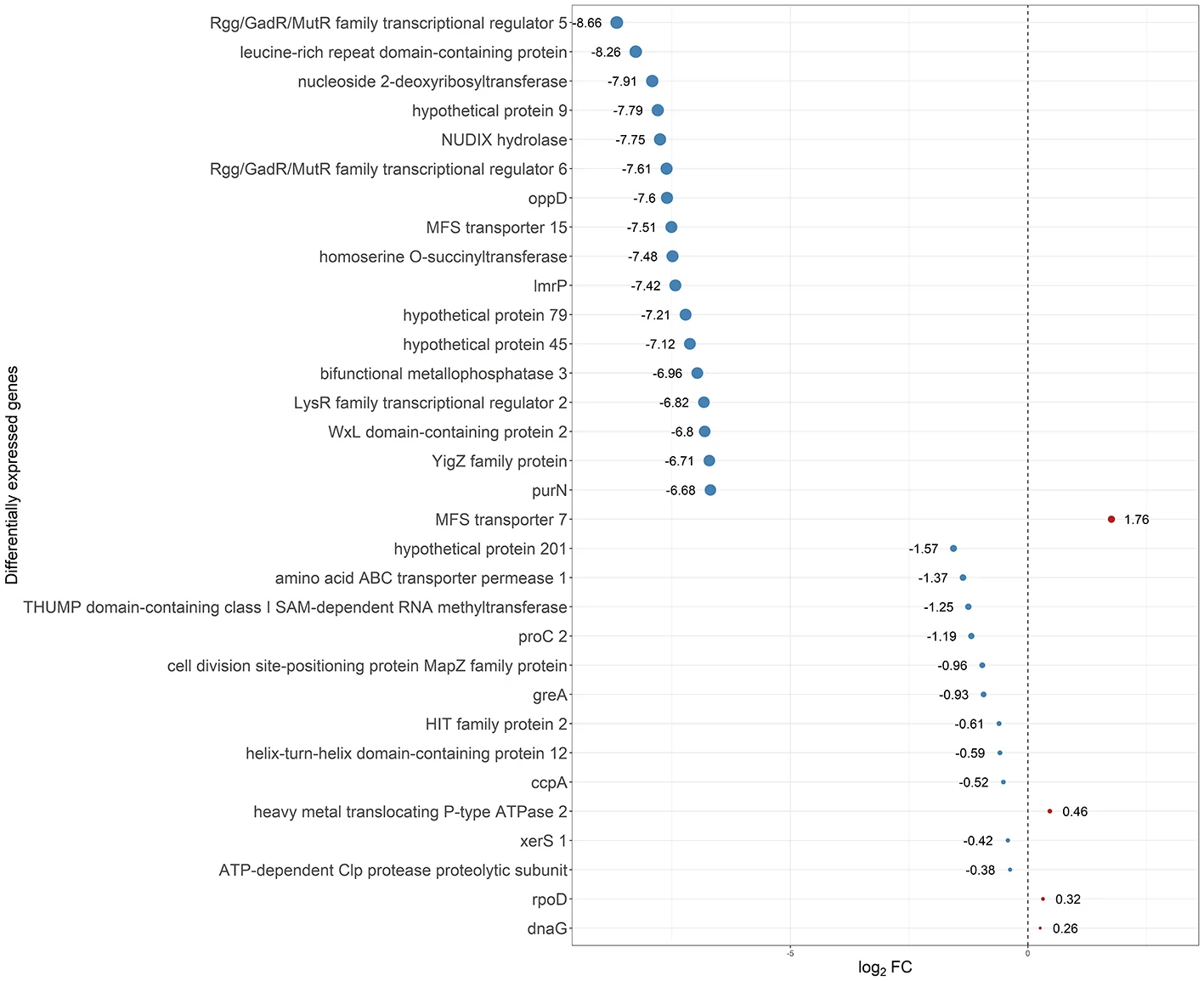
Dot plot showing significantly up and downregulated genes in the treatment group compared with the probiotic control group. Statistical significance was determined using an adjusted *p*-value threshold of < 0.1. Numbers indicate the log_2_ fold-change for each differentially expressed gene. Blue and red dots indicate the downregulated and upregulated genes, respectively. Probiotic control: BHI + *L. lactis* Treatment group: BHI + *L. lactis* + BG. Sample size for the two groups = 4 flasks per group.

### 3.3 Extracellular metabolites produced by *L. lactis*

Adopting an untargeted metabolomics approach, we detected 2,891 metabolites produced by *L. lactis* (Supplementary Table 6). Of those, 116 compounds were annotated with the highest confidence (annotation level 1), whereas 140 and 94 compounds were annotated at levels 2a and 2b, respectively. In addition, 1,267 compounds were annotated only at the annotation level 3, while 1,274 compounds remained unannotated. A total of 556 metabolites were significantly abundant in the probiotic control group compared to the BHI media, including 28 at annotation level 1, 30 at level 2a, 12 at level 2b, 220 at level 3, and 266 unannotated compounds (Supplementary Table 7). In comparison, 578 metabolites were significantly abundant in the treatment group compared to the BHI media, including 24 at annotation level 1, 32 at level 2a, 13 at level 2b, 234 at level 3, and 275 unannotated compounds (Supplementary Table 8). Both the probiotic control and treatment groups showed higher abundance (log_2_FC > 2) of metabolites such as 3,5-dimethoxyphenol, lactic acid, isethionic acid, isovalerylcarnitine, NAD^+^, orotic acid, phenoxyacetic acid, and N-acetylproline (Figure 4). Additional metabolites common to both groups included 2-hydroxycaproic acid, hypoxanthine, mevalonic acid, ornithine, pyridoxal, acetylagmatine, shikimic acid, N-lactoyl-phenylalanine, and N-acetylserine (Figure 4).

**Figure 4:**
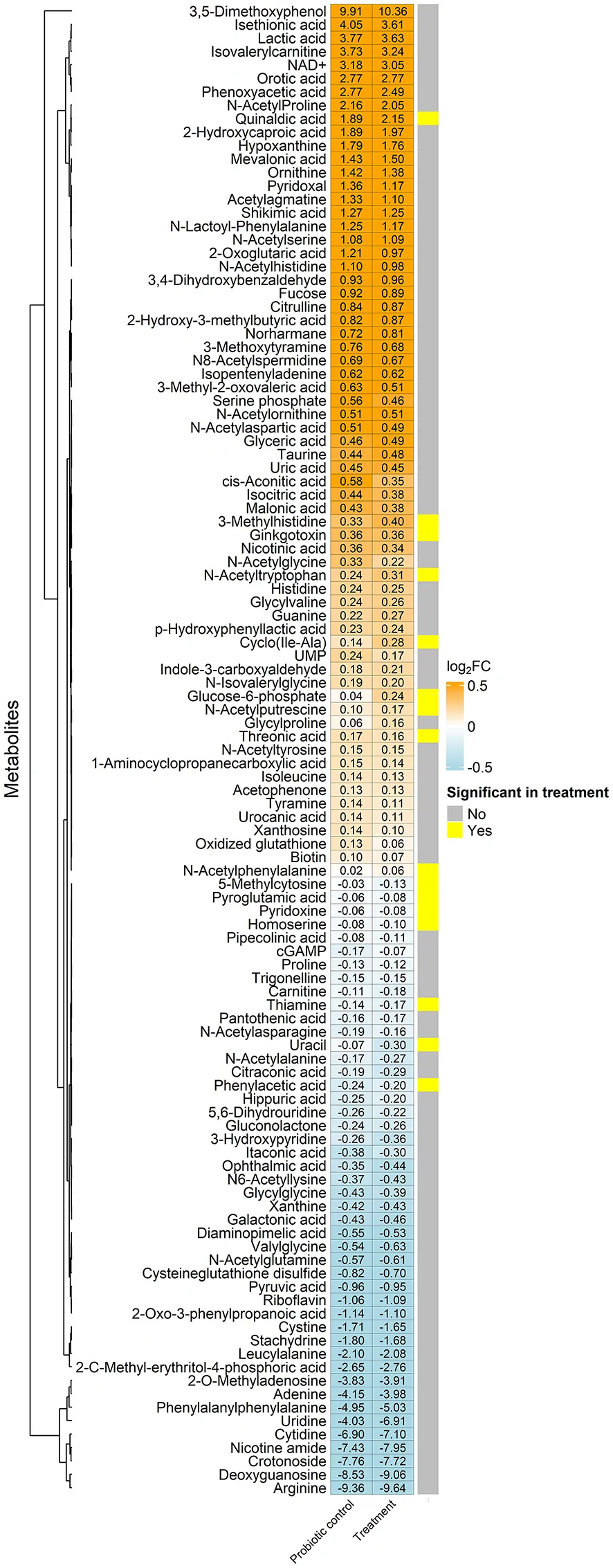
Heatmap showing extracellular metabolites that differed significantly in abundance between the probiotic control and the treatment group. “Significant in treatment” indicates metabolites significantly more abundant (*p* < 0.05) exclusively in the treatment group. Probiotic control: BHI + *L. lactis* Treatment group: BHI + *L. lactis* + BG. Sample size for the two groups = 4 flasks per group.

On the other hand, a total of 119 metabolites were significantly more abundant (*p* < 0.05) exclusively in the treatment group (based on treatment group vs. probiotic control group) including 2 at annotation level 1, 7 at level 2a, 6 at level 2b, 47 at level 3, and 57 unannotated (Supplementary Table 9). These included quinaldic acid (*p* = 0.03), 3-methylhistidine (*p* = 0.03), N-acetyltryptophan (*p* = 0.01), cyclo(Ile-Ala) (*p* = 0.03), glucose-6-phosphate (*p* = 0.01), N-acetylputrescine (*p* = 0.02), N-acetylphenylalanine (*p* = 0.02), and threonic acid (*p* = 0.03).

### 3.4 GO and KO enrichment analysis

GO enrichment analysis revealed that the upregulated DEGs were associated with DNA replication (including primer synthesis and DNA–templated replication) and RNA biosynthetic processes. At the molecular-function level, these DEGs were linked to RNA polymerase activities and ATP-dependent ion transport systems (Figure 5). In contrast, downregulated DEGs were significantly overrepresented in multiple GO terms related to L–methionine biosynthesis, including both *de novo* and homoserine–derived routes, as well as the homoserine metabolic process (Supplementary Table 10). KO enrichment with clusters of upregulated DEGs indicated the over-representation of the replication and repair (lla03030) pathway (*p* = 0.046). Other clusters (downregulated genes or metabolites with both high and low abundance) did not show any pathways with *p* < 0.1. Therefore, GSEA was performed, and two KEGG pathways (*p* < 0.1) were enriched among the expressed genes and metabolites; the sulfur metabolism (lla00920; NES = −1.62, raw *p* = 6.6 × 10^−4^, adjusted *p* = 0.07) and the ABC transporters (lla02010; NES = −1.55, raw *p* = 2.4 × 10^−^^3^, adjusted *p* = 0.1). These pathways showed negative enrichment in the treatment group (Supplementary Table 11).

**Figure 5:**
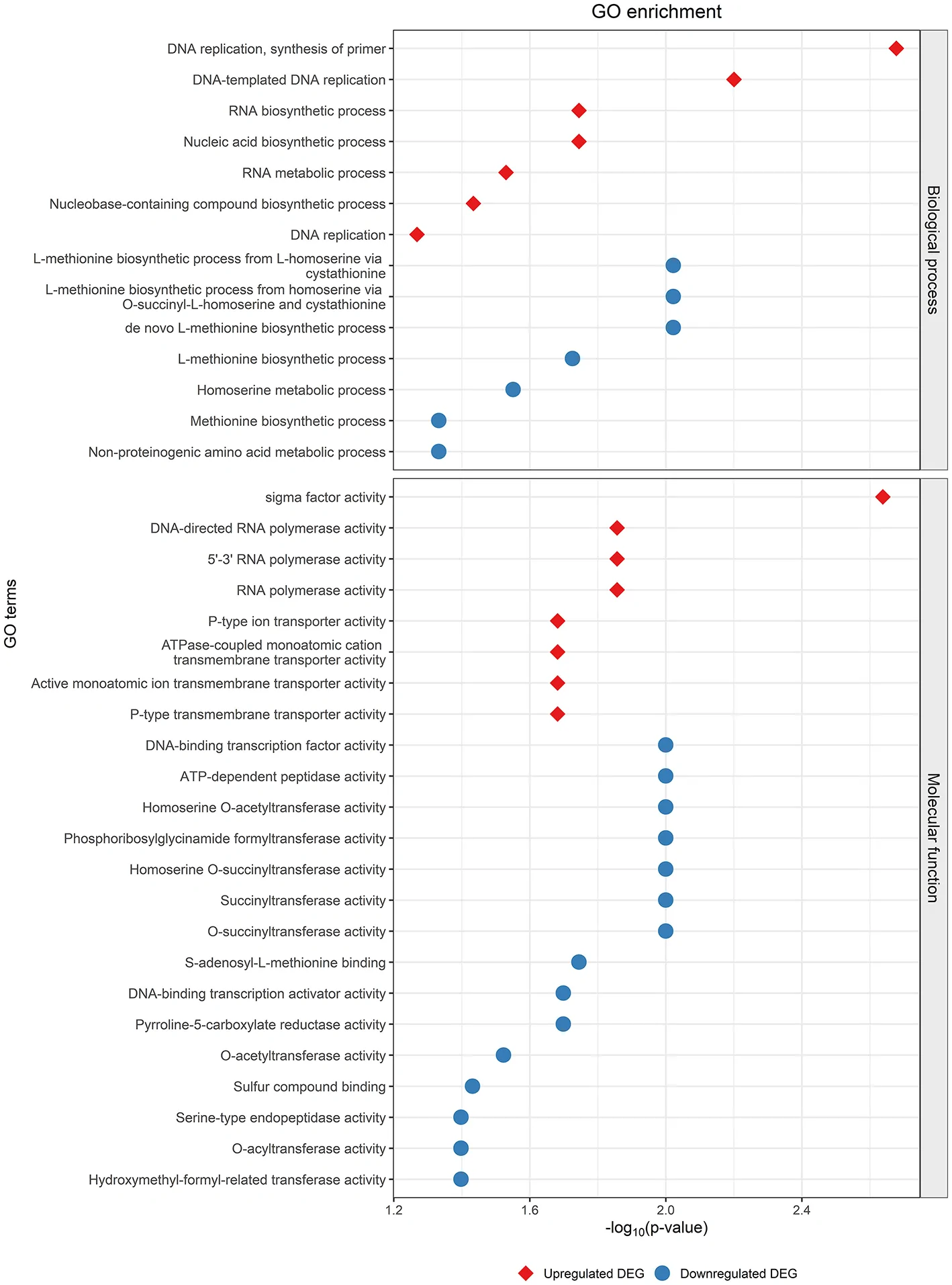
Dot plot showing significantly enriched GO terms among differentially expressed genes (*p* < 0.1). The GO term plot includes those belonging to the categories biological processing (BP) and molecular function (MF). The x-axis represents -log_10_(*p*-value). Red diamonds: enriched terms among upregulated DEGs, blue circles: enriched terms among downregulated DEGs.

## 4 Discussion

*L. lactis* is a non-sporulating, facultative anaerobic Gram-positive bacterium. This well-characterized microbial cell factory, generally recognized as safe (GRAS), is widely exploited for recombinant protein and metabolite production (Song et al., 2017). Although *L. lactis* is primarily a homolactic fermenter, its metabolism can be redirected toward alternative products through genetic engineering or environmental modulation. In the present study, a potential prebiotic substrate was employed to explore its capacity for metabolic reprogramming of *L. lactis*. The presence of β-1,3-glucan may have enabled the bacterium to initiate replication earlier, resulting in higher final cell density without inducing genes associated with quorum-sensing (e.g., *nis*) and other stress responses (e.g., *sodA, busAB, and lmrB*; Qiao et al., 2021; van der Meulen et al., 2017). The transcriptomic (ribosomal proteins, carbon catabolism relaxation associated gene, Rgg/GadR/MutR-family regulators, surface proteins, and membrane transporters) and metabolic (organic-acid pool, aromatic and tryptophan-derived metabolites, carbohydrate metabolism, amino acid metabolism, cofactor and secondary-metabolite biosynthesis) profiles suggest that β-1,3-glucan promotes an earlier transition of *L. lactis* into the stationary phase and redirects carbon flux from homolactic fermentation toward alternative metabolic pathways through coordinated reprogramming of central carbon metabolism. The observed improvement in growth performance suggests that *L. lactis* may benefit from the presence of β-1,3-glucan.

Real-time growth monitoring and flow-cytometric enumeration showed that β-1,3-glucan shortened the lag phase and accelerated the exponential growth of *L. lactis*, resulting in increased cell density. This growth pattern suggests an earlier transition into the stationary phase relative to the probiotic control group, which may be consistent with the prebiotic potential of fungal β-glucan in promoting LAB growth (Buksa et al., 2025). However, direct catabolism of the intact β-1,3-glucan is unlikely, because the genome of *L. lactis* DSM20175 lacks genes encoding exo-/endo-β-1,3-glucanases. The growth benefit may be more indirect, arising from low-molecular-weight β-1,3-glucan oligosaccharides generated during medium preparation or by extracellular degradation that may function as growth substrates (Fernandez-Julia et al., 2023). The transcriptomic and metabolomic changes further provided a mechanistic explanation of this response, going beyond a purely phenotypic description. The transcriptional signature exhibited a relaxation of carbon catabolite repression. In *L. lactis, ccpA*, encoding catabolite control protein A, represses genes involved in the uptake and utilization of non-preferred carbon sources when a preferred sugar is available (Chen et al., 2018). Downregulation of *ccpA* in the β-glucan group (treatment group) would therefore be expected to relieve this repression and allow co-utilization of alternative carbon sources (Papadimitriou et al., 2016). The metabolomic profile showed a significant increase in glucose-6-phosphate (G6P, an intermediate of glycolysis) in the treatment group. This increase suggests altered or enhanced glucose uptake and glycolytic metabolism. However, the absence of a corresponding rise in lactic acid indicates that the additional carbon flux is likely diverted from homolactic fermentation toward alternative metabolic pathways. Based on the altered organic-acid pool (e.g., pyruvic and malonic acid) as well as the changes in aromatic and tryptophan-derived metabolites (e.g., shikimic acid, *N*-acetyltryptophan, quinaldic acid, indole-3-carboxaldehyde), we infer coordinated reprogramming of central carbon metabolism (Dehhaghi et al., 2019). These findings suggest that β-1,3-glucan may enhance the metabolic efficiency of *L. lactis* by promoting a broad reprogramming and the redistribution of carbon-derived intermediates among central carbon, amino acid, and secondary-metabolite pathways, rather than directing carbon prominently toward homolactic fermentation (Averesch and Krömer, 2018; Shende et al., 2024; Wang et al., 2026). However, absolute quantification of individual compounds was not feasible in this study, necessitating verification of the findings through targeted metabolomics.

The transcriptomic analysis showed a broad downregulation of genes associated with transcriptional regulators (Rgg and LysR), surface proteins (LRR and WxL), and membrane transporters (MFS transporters, amino acid ABC transporter permease). These significantly downregulated DEGs were associated with the enrichment of the GO term “methionine biosynthetic processes.” Although methionine is an essential amino acid for protein synthesis in *L. lactis*, its biosynthesis is among the most energy-demanding metabolic pathways, resulting in a relatively low carbon yield (Hernandez-Valdes et al., 2020; Kaleta et al., 2013). This observation may indicate that the cells have shifted from an anabolic growth state toward a maintenance-oriented metabolic state. Previous studies have shown that in *L. lactis*, the transition from exponential growth to the stationary phase may begin earlier than previously assumed, with cells exhibiting increased stress resistance before reaching the stationary phase (Duwat et al., 2000). In our study, the treatment group culture reached a substantially higher cell density (1.2 × 10^8^ cells/mL) than the probiotic control group, suggesting that the cells may have entered an early transition phase and initiated stress adaptation. In parallel, we observed a strong downregulation of Rgg/GadR/MutR-family regulators, which may reflect alterations in regulatory networks involved in stress responses, acid tolerance, and quorum-sensing signaling (Lingeswaran et al., 2020; Yogeswara et al., 2020). Although canonical quorum-sensing signaling molecules of Gram-positive bacteria such as autoinducing peptides (Das et al., 2026) were not directly quantified, untargeted metabolomics revealed an increased abundance of cyclo(Ile-Ala), a cyclic dipeptide with a diketopiperazine structure. The biological function of this metabolite in *L. lactis* is unknown, and no direct association with quorum-sensing systems has been reported (Hatanaka et al., 2020). Nevertheless, Rgg transcriptional regulators are known to bind to peptide pheromones and regulate, among other processes, cell-cell communication (Das et al., 2026). During the presumed stress adaptation and maintenance state, we observed a downregulation of *lmrP*, encoding a drug/H^+^ antiporter, plausibly indicating a reduced demand for efflux activity under favorable growth conditions (Papadimitriou et al., 2016). Likewise, suppression of genes encoding LRR and WxL domain-containing proteins further suggests cell-wall remodeling and reduced investment in non-essential surface structures, allowing cellular resources to be redirected toward cell maintenance-associated processes (Desvaux et al., 2018; Foltran et al., 2024; Galloway-Peña et al., 2015). Although these changes are indicative of a progression toward a maintenance-oriented physiological state, the slight upregulation of *dnaG*, encoding the bacterial primase required for DNA replication, and *rpoD*, encoding the primary housekeeping sigma factor, suggests that the cells have not yet fully exited the growth stage. Instead, these genes may represent a transient transcriptional state during the transition from exponential growth to stationary phase, before complete downregulation of core growth-associated genes (Miura et al., 2015; Park et al., 2024; Zhou et al., 2017).

Although fewer in number, the upregulated DEGs provide important insights into cellular metabolic adjustments. The heavy metal-translocating P-type ATPase, a type of cation transporter, together with the upward trends in *feoB* (Fe^2+^ transporter) and a “cation diffusion facilitator transporter”, may indicate altered metal homeostasis (Chandrangsu et al., 2017; Sestok et al., 2022). Metal homeostasis critically determines the availability of cofactors required by many essential enzymes and this process maintains the intracellular metal concentrations (Kunkle and Skaar, 2023). Several studies have reported that bacterial cyclic β-1,2-glucans, cyclic β-1,3-1,6-glucans, and cereal and fungal β-1,3-1,4-glucans possess attenuated metal-binding (chelation-like) properties, which may modulate mineral bioavailability (Ashraf Khan et al., 2017; Javvadi et al., 2017; Marasca et al., 2022; Visweswar et al., 2018). β-1,3-glucan from *E. gracilis* differs structurally from these β-glucans. Unlike cyclic β-1,3-glucans, which form highly cation-selective inclusion complexes, its linear structure may permit weak interactions with metal ions via hydroxyl groups, exhibiting chelation-like behavior that could potentially influence metal availability for biosynthesis while limiting toxicity (Kuhaudomlarp et al., 2018; Visweswar et al., 2018). Nevertheless, this hypothesis needs to be tested experimentally.

The small sample size in the transcriptomic and metabolomic analysis could have contributed to the relatively low number of significantly differentially expressed genes and differentially abundant metabolites. The transcriptomic dataset was not generated using the culture collection strain DSM20175, the genome of which is available in NCBI as LMG7760. Instead, the dataset presented in this manuscript was based on genome assembly ASM317683v1, which provided a greater number of gene annotations, thereby facilitating better pathway enrichment and enabling an appropriate interpretation of the physiological state at the transition phase of *L. lactis* into its stationary phase.

Finally, the findings of this study suggest potential applications in synbiotic formulation and industrial fermentation processes, as β-glucan supplementation may support increased biomass production and the formation of bioactive secondary metabolites such as shikimic acid, quinaldic acid, and a range of small peptides (Buksa et al., 2025; Fidan et al., 2022; Wang et al., 2021). Given the known immunomodulatory properties of β-glucans, this microalgal glucan could be explored for use in functional food applications (Rossella et al., 2017).

## 5 Conclusion

In this study, *E. gracilis*-derived β-1,3-glucan supplementation significantly increased the growth rate and final cell density of *L. lactis* DSM20175 without a corresponding rise in extracellular lactic acid. Transcriptomic profiling revealed a small, predominantly repressive response (23 of 26 significant DEGs downregulated), while untargeted metabolomics showed elevated aromatic and tryptophan-derived metabolites as well as small peptides in the treatment group. Together, these results demonstrate that β-1,3-glucan acts as a growth substrate for *L. lactis*, increasing biomass and diversifying secondary-metabolite output rather than fermentative acid production. The accompanying shifts in carbon-catabolite, translational, and stress-related regulators are consistent with an accelerated growth-phase transition toward a more resource-efficient state. However, these mechanistic links require targeted functional validation, which would deepen our understanding of prebiotic-probiotic interactions and provide a foundation for the development of optimized synbiotic formulations.

## Data Availability

The datasets presented in this study can be found in online repositories. The names of the repository/repositories and accession number(s) can be found below: https://www.ncbi.nlm.nih.gov/, PRJNA1418359.
